# Remote Working in a Public Bureaucracy: Redeveloping Practices of Managerial Control When Out of Sight

**DOI:** 10.3389/fpsyg.2021.606375

**Published:** 2021-11-25

**Authors:** Martina Hartner-Tiefenthaler, Melanie Goisauf, Cornelia Gerdenitsch, Sabine T. Koeszegi

**Affiliations:** ^1^Institute of Management Science, Labor Science and Organization, Vienna University of Technology, Vienna, Austria; ^2^Department of Science and Technology Studies, University of Vienna, Vienna, Austria; ^3^Austrian Institute of Technology (AIT), Vienna, Austria

**Keywords:** new ways of working, managerial control, institutional logics, interview study, praxeological analytic approach, public bureaucracy

## Abstract

This article examines managerial control practices in a public bureaucracy at the moment of introducing remote work as part with a *new ways of working* (NWW) project. The qualitative study builds on 38 interviews with supervisors and subordinates conducted before the advent of COVID-19. By interpreting interviewees’ conversations about current and anticipated future work practices in the changing work setting, we reveal tacit and hidden practices of managerial control that are currently prevalent in many organizations introducing remote working. Three constitutive moments of the organization’s transformation to NWW are analytically distinguished: (i) how implicit becomes explicit, (ii) how collective becomes self, and (iii) how personal becomes impersonal. Our findings emphasize that the transition to NWW must take into account prevailing institutional logics and must reconnect to a fundamental and often neglected question: What does *doing work* mean within the particular organization? Negotiating this fundamental question might help to overcome supervisors’ uncertainties about managerial control and provide clarity to subordinates about what is expected from them while working remotely. Finally, we discuss how the transition to NWW may serve as both an opportunity and a potential threat to established organizational practices while highlighting the challenge supervisors face when the institutional logics conflict with remote working.

## Introduction

Recently, remote working has been rapidly introduced in many organizations all over the world due to the COVID-19 pandemic. Since the threat of spreading COVID-19 in the office is still present, many organizations are considering a transformation toward more “novel” work practices (Von Blazekovic, 2020), often subsumed as “new ways of working” (NWW). NWW absorb perspectives previously described as “telecommuting” (Feldman and Gainey, 1997) or “schedule control” (Kelly et al., 2011) and are characterized by greater flexibility in work time and space and also by an increased use of information and communication technologies (Demerouti et al., 2014). Even before the COVID-19 pandemic, the adoption of NWW was steadily increasing (European Foundation for the Improvement of Living and Working Conditions, 2015) reflected in national laws that stipulate the right to request flexible working arrangements (e.g., the Flexible Working Act in Netherlands, Overheid, 2016 or the Flexible Working Regulations the United Kingdom, The National Archives, 2014). Existing literature highlights the need to re-align managerial control when employees’ work time—and especially work space—has become more flexible (Kurland and Cooper, 2002; Taskin and Edwards, 2007; Field and Chan, 2018). Although there seems to be a consensus that managerial control practices must be adapted for NWW (Kurland and Cooper, 2002; Taskin and Edwards, 2007), little is known about how this process can be organized in a public bureaucracy. This paper follows a praxeological research approach (see overview in Reckwitz, 2002) to understand how the material, spatial, and embodied practices are meant to be enacted at new and dispersed sites (Schatzki, 2005), and how this is challenged by pre-existing assumptions about how work is done (Ashforth et al., 2000).

In general, transformations of work practices have a profound impact on management practices and are accompanied by a comprehensive adaptation process in organizations (Kingma, 2019). When implementing remote working, organizations must re-think their work and managerial control practices—in particular when they have previously relied on the presence and visibility of employees (Bailey and Kurland, 2002; Kurland and Cooper, 2002; Dimitrova, 2003; Taskin and Edwards, 2007; Sewell and Taskin, 2015; Peters et al., 2016). Managerial control practices are fundamental in organizations as they coordinate employees’ efforts, enable agreement between managers at different hierarchy levels, serve as a source of motivation by setting up an incentive system, and function as signals to trigger necessary interventions by management (Goold and Quinn, 1990). With the advance of communication technologies, managerial control is inherently connected to technology (Orlikowski and Scott, 2008; De Vaujany and Vaast, 2014). Technology can be used to remotely monitor employees’ performance (Kolb and Aiello, 1996; Alder, 2001) by such methods as controlling key strokes, computer time accounting, global positioning system (GPS) surveillance, or telephone call monitoring. Due to the high reliance on technology in daily work practices, organizations can continuously observe, record, or analyze information on employees’ behavior. Electronic performance monitoring results in benefits such as increased task performance (Aiello and Kolb, 1995; Bhave, 2014) but also negative effects for individual workers such as perceived stress (Amick and Smith, 1992) and reduced job satisfaction (Jeske and Santuzzi, 2015). When face-to-face contacts between supervisors and subordinates are reduced, the issue of control is amplified and direct monitoring is no longer feasible (Kurland and Cooper, 2002; Sewell and Taskin, 2015). Sociological approaches to work and control (see overview in Thompson and van den Broek, 2010) are often influenced by Foucauldian theory and focus on managerial practices as well as surveillance and discipline, also in connection to new technologies (Sewell and Wilkinson, 1992; Sewell, 1998).

In NWW, employees are encouraged to decide autonomously when and where to work based on personal preferences as well as organizational requirements. However, employees’ increased autonomy in NWW environments challenges organizations to ensure that individuals’ activities remain aligned with organizational strategies (Zafari et al., 2019). Thus, the implementation of NWW is often accompanied by performance measurements based on employees’ output (Taskin and Edwards, 2007). Output can be measured in terms of goals, objectives, budgets, or deliverables (Eisenhardt, 1985; Ocasio and Wohlgezogen, 2010). When output control is in place, time and place of work become irrelevant as achievement is assessed based on performance results rather than behavior (Kurland and Cooper, 2002). Thus, prevailing output control would require low adaptation of control practices when transitioning to NWW. However, a strong focus on output control has also been criticized due to its shifting of responsibility for work from the employer onto the employee (Voß and Pongratz, 1998).

In addition to output control, there are informal control practices, which represent a more subtle form of control (Etzioni, 1964; Eisenhardt, 1985; Dermer, 1988; Barker, 1993; Kunda, 1995; Alvesson and Willmott, 2002; Kärreman and Alvesson, 2004; Fleming and Sturdy, 2011). Informal control is exerted *via* rituals and ceremonies based on reciprocity, legitimate authority, common values, beliefs, and traditions (*cf*., “clan control” by Ouchi, 1980). The underlying rationale is that, in contrast to output control, informal control attempts to manage employees’ beliefs, norms, and interpretations in an implicit manner (Kärreman and Alvesson, 2004). It is seen as a very powerful form of control in remote settings as workers are committed to the organization (de Menezes and Kelliher, 2017). However, remote workers might also be more stressed due to the intensification of work as for them it is easier to work harder or longer than for their non-remote counterparts (Kelliher and Anderson, 2010). Enabled by digital technologies, remote work is also performed outside traditional work hours and workplaces (Mullan and Wajcman, 2019). Consequently, it blurs the boundaries between work and non-work (Chen and Nath, 2005; Gottschall and Voß, 2005), which lead to deleterious effects on employees’ well-being (e.g., Chesley, 2005; Russell et al., 2009; Schlachter et al., 2018).

In public bureaucracies, managerial control is generally closely linked to direct monitoring and surveillance of employees’ behaviors (Ouchi, 1977). It is mostly based on formal principles (Diefenbach and Sillince, 2011) that rely on explicitly formulated rules and routines (Styhre, 2008; Ocasio and Wohlgezogen, 2010; Merchant and Van der Stede, 2017). Daniels et al. (2001, p. 1171) suggest that remote work is less likely “where bureaucratic control and stability are valued.” Empirical research shows that in public-sector organizations, the adaptation of control practices toward more flexibilization is vulnerable to failure, but it *can* be managed successfully (Taskin and Edwards, 2007; Taskin, 2010). Taskin and Edwards (2007) identified the following seven elements relevant for establishing remote working in a public organization: commitment from top management, participation of trade union representatives, no individual performance statistics, written agreement defining frequency of remote working, supervisors retaining authority to permit or deny work from home, frequent meetings to inform employees about projects, and implementing the transformation as part of a global strategy. In line with these elements, the organization under study refrains from “performance tracking on an individual level” which, however, contradicts the common practice of output control in remote settings (Kurland and Egan, 1999; Felstead et al., 2003). Thus, the transformation to NWW might contradict the prevailing institutional logics requiring managing competing logics (Reay and Hinings, 2009). “Institutional logics” are understood as “socially constructed, historical patterns of cultural symbols and material practices, assumptions, values and beliefs by which individuals produce and reproduce their material subsistence, organize time and space, and provide meaning to their daily activity” (Thornton et al., 2012, p. 50). Therefore, changing the location of work involves more than providing adequate tools and technology—supervisors must make sense of what kinds of managerial control practices are considered legitimate (or illegitimate) when employees work remotely. Therefore, we argue that supervisors need organizational support to shape their leadership practices and manage competing logics.

Our study sheds light on how the transition to NWW is negotiated by the organizational actors (supervisors and subordinates) in a public bureaucracy. We use a praxeological approach to understand how organizational actors handle conflicting demands from competing logics (Reay and Hinings, 2009; Bjerregaard, 2011) and emphasize the importance of taking into account the underlying institutional logics (Thornton et al., 2012; Thorén et al., 2018) when implementing NWW, since the institutional logics may be reshaped *via* adapted control practices (Glynn, 2013). Little is known about the adaptation of prevailing work practices and the individual attributions during this process (Delanoeije and Verbruggen, 2019; Wessels et al., 2019). Using 38 interviews with supervisors and subordinates in a public bureaucracy, we analyze imaginings of future work settings against the backdrop of current practice and investigate the prevailing assumptions about managerial control. Most existing studies evaluated the adaptation process *after* the implementation of “new” working practices, whereas we analyze an earlier moment in the implementation process, when new working practices are still in transition and open to debate by the actors involved. Capturing paradoxical situations and tracing practices before they become hidden and tacit within daily routines allow us to derive knowledge how organizations should manage their transition toward NWW.

The paper contributes to the debate surrounding re-regulation of managerial control for NWW by highlighting the role of institutional logics for a successful implementation of NWW. The COVID-19 pandemic forced many organizations to offer remote working for their employees—regardless of whether their institutional logics support NWW. Recently, some restrictive measures imposed in response to COVID-19 have been rolled back, but a critical mass of employees now ask for the possibility to continue working remotely in the future (Von Blazekovic, 2020). Investigating how organizational actors negotiate the implementation of NWW allows us to uncover adaptation strategies for remote work in organizations that heavily rely on employees’ physical presence at the work site. For deciding upon the extent of remote work and considering the possibilities of NWW, it is necessary to understand potential tensions in order to picture ideas how the transition toward NWW could be rolled out.

In our analysis, we identify three constitutive moments of transformation and elaborate upon: (i) how implicit becomes explicit, (ii) how collective becomes self, and (iii) how personal becomes impersonal. Based on this analysis, we argue that the implementation of NWW requires taking into account the prevailing institutional logics of the organization. In particular, we raise employees’ awareness of hidden electronic performance monitoring, even in an organization that officially eschews individual performance measurement. Our analysis uncovers the perception among supervisors that employees need to be monitored and surveilled. This also reveals supervisors’ insecurities about managerial control when traditional workplace interaction ends. In addition to the role of supervisors and their uncertainties about leadership, we highlight the need to clarify what *doing work* means in the respective organization when transforming toward NWW. We understand doing work as the performative act on how to *do* work and how this is reconstructed and routinized by the actors involved.

## Materials and Methods

### Research Setting: The Case of PubConsult

Our case study focuses on the early stage of transformation to NWW at PubConsult,^1^ a public bureaucracy in Austria. Despite the general trajectory of Austrian implementation of the phenomenon known as “new public management,” the dominant logic in Austrian public bureaucratic organizations is still “characterized by a strong emphasis on processes, rules, and directives together with a relatively high amount of informality and a primarily internal orientation” (Meyer and Hammerschmid, 2006, p. 1003).

The culture of PubConsult follows bureaucratic principles, but also places a very strong emphasis on values such as solidarity and justice. Similar to public organizations (Boyne, 2002; Taskin and Edwards, 2007), PubConsult is characterized by steep hierarchies, a reliance on formal rules and standard operating procedures, and a norm of physical presence and on-site working. Performance management at PubConsult consists of monitoring employees’ physical presence (and visibility) on-site, defined by a time stamp at the entrance of the office building. PubConsult offers stable, secure jobs and is financed by obligatory contributions from its members. At PubConsult, turnover of staff is low and layoffs are extremely rare, which is reflected in our sample. On average, interviewees had worked within the organization for 17.63years (*SD*=8.98).

Prompted by the upcoming relocation of its head office to a nearby city, PubConsult launched a project to introduce NWW that aimed to compensate for the longer commuting times that would affect the majority of employees. Although PubConsult aimed to encompass various aspects of NWW, such as digitalization of work documents, upgrading working devices, and an activity-based flexible office (Wohlers and Hertel, 2017), the most evident change was that, as part of the NWW project, remote working was offered to all employees. A new employment agreement stipulated that employees could work up to 20h per week outside the office when prior notice was given. During this remote working time, employees were required to be available either *via* mobile phone or *via* an online communication tool. The agreement further stipulated that supervisors could request a videoconference (i.e., “turning on the camera”) when they considered it necessary. This issue caused heated debates among employees about control and trust of leaders since the regulation was also perceived as a sign of distrust. Drawing on these debates, we analyze the managerial control practices and shed light on the prevailing institutional logics.

### Research Approach: Focus on Practices

A practice theoretical lens is used as “sensitizing concept” (Blumer, 1954) to explore PubConsult’s practices, by which individuals (re)produce the material, temporal, and spatial arrangements. A “practice” is understood as comprising a set of actions, i.e., “doings and sayings” that are performed as “embodied, materially mediated arrays of human activity centrally organized around shared practical understandings” (Schatzki, 2006, p. 2). These practices are organized in routines—for example, how work is started and finished, or how work is conducted in the office—and take place within spatial contexts, that is, at a workplace within an organization. Following this understanding, organizations are conceived as *sites* that are constituted by, and in a bundle of, practices and material arrangements (Schatzki, 2005). The nature of actions performed is determined by these sites, which are in turn constituted through practices. *Materiality*, comprising the performative power of (technological) artifacts, plays a crucial role in how practices are implemented and how practices of control are inscribed in spatial settings and objects of daily work routines within an organization. Following the previous literature (Bjerregaard, 2011), this article takes a praxeological approach to understand the prevailing institutional logics of a public bureaucracy.

PubConsult constitutes a unique case for the empirical investigation, facilitating an analysis of processes that are usually hidden at the “backstage” (Goffman, 1956) of organizations. Aside from its strong mission to protect employees’ rights, PubConsult’s work routines seem rather typical for public bureaucratic organizations. The transition to NWW at PubConsult was accompanied by heated internal discussions, disruptions, and conflicting expectations regarding changes to established work routines despite its intention to improve employees’ working conditions. This case, therefore, constitutes an empirically rich resource to show how the introduction of NWW challenges prevailing managerial control practices and reveals prevailing institutional logics. In brief, institutional logics “become manifest in practices, and, in turn, practices render logics transparent” (Glynn, 2013, p. 493).

### Data Collection and Analysis

The findings are based on a sample of 38 semi-structured interviews with supervisors and subordinates that were conducted at the beginning of the process of transition to NWW and were contextualized with internal documents and field observations. Interviewees were selected in cooperation with the organizational development department. The selection was critically reflected upon during the sampling process. Interviewees differed in age, gender, tenure, department affiliation, and hierarchical position (20 supervisors and 18 subordinates). The interviews were conducted in German in a rented room nearby the organization to make it easier for interviewees to speak freely. Participation was voluntary, and anonymity was assured in the treatment of the data. Interviews lasted approximately 1h and were recorded and transcribed.

The interviews had narrative elements, and interviewees were encouraged to elaborate on their answers. They received the opportunity to disclose their opinions without much interruption from the interviewer. All interviews started with a description of the interviewees’ current position in the organization and the tasks involved in that position. Subsequently, interviewees were invited to describe their perceived organizational culture using thirteen stimulus words such as trust, authority, social justice, control, and feedback. Finally, the interviews concluded with questions about the forthcoming change toward NWW. Memos summarizing the content of the interviews enriched by contextual information about the interview situation were produced for each interview.

Our research team visited the organization several times, ate lunch in the cafeteria commonly used by staff, and observed interactions in the entrance hall. One of the authors was involved in an assigned project to evaluate the NWW transformation process and participated in staff meetings. Furthermore, the results of an internal employee survey, the employment agreement, and press releases were available to the research team. The analysis presented in this paper consists of the interview transcripts, as the observations were only used to supplement the interview data.

The data were analyzed using interpretative methodology and a grounded theory approach (Charmaz, 2006). In doing so, selected sequences were interpreted line by line by a moderated group of three to six interpreters reflecting on the following questions: What is being said on the content level? What underlying assumptions and mental models could have provoked the interviewee to say what she/he has said? What kind of language is used and how is it used? What actors are involved and what conclusions can be made regarding the structure and dynamic of the social system (i.e., the organization)? Step by step, more narratives were included in this fine-grained analysis until theoretical saturation was reached. In this process, theoretical concepts (in particular from Bourdieu, Goffman, and Foucault) were used as inspiring tools and helped us to describe the underlying meaning in the material.

## Findings: Enacting Managerial Control in Transition

The described work practices at PubConsult are not solely enactments of—or responses to—NWW, but are rather already established, inscribed, and incorporated practices reflecting the bureaucratic logic that is being adapted to changing work arrangements. NWW advocates autonomy and responsibility for employees, which may contradict bureaucratic principles that rely on rules and formalized procedures. Analyzing managerial control practices at PubConsult prior to the transformation process captures paradoxical situations and tensions. The analysis carves out how established practices of control have been inscribed in daily working routines but are challenged and brought to the surface by the transformation toward NWW. Three constitutive moments of the transformation are revealed at the start of the transformation process: (i) how implicit becomes explicit, (ii) how collective becomes self, and (iii) how personal becomes impersonal.

### Making Implicit Control Practices Explicit

With the first constitutive moment, this article traces how implicit control practices are made explicit in descriptions of daily working routines. First, the interviewees denied the prevalence of direct monitoring practices. For example, two subordinates emphasized that “Monitoring of actual work is non-existent” and “Monitoring someone extremely closely—I am not really aware that this exists” (Subordinates). Overall, most interviewees took a rather critical view of monitoring as a managerial control practice as it contradicts deeply anchored core values of the organization, such as being intrinsically loyal and collegial. A deeper analysis, however, reveals descriptions of monitoring in many narratives, even before the interviewer explicitly asked about it: They are inscribed in acts of entering or leaving the workplace and “being at work.” Hence, interviewees’ conception of managerial control was mainly restricted to monitoring physical presence and visibility. However, control was exercised rather implicitly, inscribed in signaling practices (Goffman, 1956) within daily working routines, as illustrated in the following quote from a supervisor:

So, if someone is physically here, then he is here and then I see what he is doing. Otherwise, I actually have to look it up now [in NWW], so to speak, as a form of monitoring, when it is wanted, what has been accomplished. In accounting that is easily possible because I can check what anybody has entered. But I need to take an additional effort to check it; otherwise during the day, yes, I know what has been done and has been said and I do not need to check it.

*Interviewer: Okay. But if the person is present, you still can’t be sure that the work has been done?*

People usually say goodbye in the evening. Before leaving, it is common for my team to come up to me and inform me such as “I’m leaving now.” That way I know, and then I ask them “Is that all, is everything done? Yeah, good. What we agreed on?” Then I *hear* it directly. (Supervisor)

The beginning of the quote points out how physical presence is interpreted as “doing work.” Being “there” and being able to see what somebody is doing is considered sufficient proof of doing work. While actual work performance may happen out of the supervisor’s sight (even while in the office), the ritualized way of leaving the workspace enables this supervisor to check whether the work has been completed based on the employees’ words. This narrative shows how “doing work” is signaled through small acts, such as words, which points to the power of the symbolic in exercising control and discipline (Bourdieu, 1991); it also indicates that these signals have been tacitly acquired through a socialization process, facilitated by the generally long tenure of the staff. The implementation of NWW disrupts the well-established modes of knowing how work is performed, as shown in the next quote, where the supervisor was explicitly asked about monitoring practices. Here, the spatiality of work is again tied to its temporality:

Control has so far been conducted by the time clock, meaning that when an employee enters the building he is meant to be “productive” and when he leaves the building, work ends. Strangely enough, when they [the management] started to let people work from home, suddenly questions emerged about how to monitor whether employees are *really* working [at home]. (Supervisor)

The time clock marks the turning point from “not working” to “working,” or, as the interviewee puts it more precisely, being “productive.” Entering and leaving the building, seeing or hearing, and being seen or heard at the beginning and end of the workday constitute a meaningful part of the practice of “being at work” and “doing work.”

Supervisors fear losing control due to their lack of knowledge about how to monitor behavior over a distance, while subordinates are uncertain about how to signal that they are “doing work” by means other than their physical presence. Employees anticipate the need of constantly signaling that they are “doing work” throughout their working hours:

You have to give an account of each minute. You have to be online permanently when you are in home office (Subordinate).

These signaling acts are perceived as necessary, despite the use of an online login tool that tracks employees’ work time. Although the replacing artifact could be considered to be equivalent to the time clock, it is not considered sufficient to define “doing work,” perhaps due to the potential for abuse. Thus, the underlying question becomes: What is “doing work” at PubConsult after all? In contrast to work at the office, supervisors ask for a more precise definition and documentation of tasks during home office:

Clearly defined work activities, which of course have to be checked afterwards in terms of whether they have been carried out. (Supervisor)

Reporting and transparency of individual performance become more central and affect the structure of work itself. Within this, control practices based on individual output are implemented implicitly by the supervisors, even though these practices actually contradict PubConsult’s supposedly untouchable core values of solidarity and collegiality.

The perceived shift from behavior control to output control in NWW while retaining core organizational values should not be understood as one form of control replacing another. Rather, it needs to be understood as uncovering deeply anchored tacit logics about how to carry out work, which must be transferred and adapted to the new setting. The lack of concrete practical knowledge on how to perform work creates a situation that conflicts with what the organization stands for. How “doing work” can be translated into new control practices must be negotiated. In other words, supervisors do not know how to monitor performance without performing (excessive) surveillance, and subordinates do not know how to signal “doing work” beyond the material, spatial, and temporal boundaries of the familiar work site. This entails a substantial change in the understanding of work and managerial control, as shown with the second constitutive moment.

### Collective Becomes Self

The second constitutive element shows how the transition to NWW at PubConsult accompanies a shift from a collective to an individualized form of control that manifests itself in a more self-disciplining work practice. In NWW, this self-disciplining process usually can be managed by exerting output control on the employees, but since individual output control is—officially—not possible at PubConsult, different mechanisms must be applied. The shift resonates strongly with Foucault’s (1977) description of the effectiveness of power and control: the disciplining of the self. Therefore, his theses act as “sensitizing concepts” (Blumer, 1954) in this analysis, and his terminology is utilized to bring clarity to our findings.

Foucault’s theory helped us uncover three major conflicts that arose in connection with control and NWW in the interviews: fear of control through constant unidirectional surveillance, that is, the *panopticon*; the examination and norming of individual output; and the fragmentation of groups into self-disciplining individuals.

The anticipated need to “account for each minute” while remote working is connected to a fear of constant surveillance. This is best captured in discussions about installing a camera on one’s computer, as is explicitly stipulated in PubConsult’s employment agreement with regard to “reachability”: “Supervisors can request the use of video software in case the task requires it. PubConsult will provide a camera if the home computer is being used and no camera is available.” The impression of constant observation invoked by the reference to the camera raised the issue of distrust and the rupture of core organizational values such as collegiality and solidarity. Most of all, it stands in complete contradiction with the familiar practice of how to signal “doing work.” The impression of constant observation created by the camera translates into perceived pressure to constantly signal “doing work.” This is linked to the fear that a missed video call might be interpreted as not being on duty, as illustrated in the following quote:

Um, but just because I am not logged on in […] doesn’t mean I’m not working, right? Or because I have no activity on my computer, doesn’t mean that I am not doing work, because I can be […] reading, right? So […] I think it’s a pity, uhm […] monitoring is a little more intense than necessary, I think. (Subordinate)

So there is still this thought of monitoring. I can be sure, I can chat with them any time within the time frame as they must sit in front of his computer. So, I think that the idea of monitoring is still very prevalent and trust seems to be missing. (Supervisor)

Foucault describes how the creation of the impression of constant observation makes disciplining so effective. Monitoring is condensed into a “gaze” that combines the acts of seeing and being seen (see Brivot and Gendron, 2011). When actors feel like they are being watched—even when they are not—conformity is more likely. In NWW, creating the impression of being constantly watched is closely connected to technology. The time clock at the entrance to the building is replaced by logging onto an online communication tool that is constantly sending information about who is online—and thus on duty. Electronic ways of signaling work behavior arise, replacing the previously more informal and symbolic form of practiced control. Despite management’s communicated rejection of individual performance monitoring, it is now anticipated that IT systems could be used by supervisors as an individualized form of output control. In this respect, the camera—understood as a symbol of control, surveillance and mistrust—brings issues of control to the forefront.

This in turn brings us to the second mechanism of control, according to Foucault, relating to the assumed shift from externally directed performance communication (outside the organization) to individual performance indicators (inside the organization). Organizational performance indicators such as statistics and reports about total consultations were used for external scrutiny. They have been in place to prove that assigned resources have been spent appropriately by the organization. Due to the transition to NWW, supervisors perceive a loss of control over subordinates’ behavior and the need for control rises. Thus, quantifying output is no longer exclusively used as an outward-directed mean, but also as a tool to monitor individuals’ work performance within the organization—the reversal of the gaze and the rise of individual examination, in Foucault’s terms. Translating the gaze into contemporary tools of technology, this new requirement is pointedly articulated by one supervisor: “Basically, I allow only those tasks to be worked on from home that are carried out and documented in an Excel sheet” (Supervisor). Similarly, another supervisor mentions:

The fact that I don’t have every employee present in my office, this is a huge change, because it is different whether I meet my subordinates in the corridor or I have to talk with them two days beforehand about what they will be working on at home, I have to reflect on what they [the subordinate] will be doing at home. (Supervisor)

Making the individual more visible, and thus measurable—and, in doing so, facilitating the self-disciplining of employees at sites out of sight—follows control practices that were implicitly established before the implementation of NWW, which brings us to the third mechanism of control described by Foucault. A recurrent pattern in the interviews is that interviewees distance themselves from a discursively constructed group of “non-workers.” This group is characterized as lacking the will to “do work” in the sense described in the first constitutive moment. It is feared that these “non-workers” will be able to hide more easily when out of sight and thus take advantage of the organization’s goodwill toward its employees, as individual sanctioning mechanisms are not in place.

There are people who […], I don’t know *what* they are doing. […] I think it’s a pity, because, at its core, I really believe in the organization and I know many great people here. (Subordinate)

In our interpretation, the construction of the group of “non-workers” has the following functions: It serves as a means of positively distinguishing oneself from this group of “non-workers,” and it marks an internal boundary, thus demonstrating the internalization of organizational values and one’s belonging to the group of committed employees who are “doing work.” This seems to be important because objectified criteria, such as individual performance ratings, are non-existent. With the separation of groups into individuals working at dispersed sites, this social categorization would lose its effectiveness. Instead, self-disciplining *via* the impression of constant surveillance, as well as the examination of numerical measures of individual work output, replaces this mode of informal control, which is focused mainly on the process itself, by making the individual—not the group—more visible. Furthermore, the constitutive shift from collective to self fundamentally contradicts the logics at PubConsult as it understands itself as an organization that represents the interests and rights of its members as a collective. Indeed, PubConsult derives its power from being a collective movement. The shift of focus onto the individual fundamentally contradicts this idea und undermines this purpose.

### Personal Becomes Impersonal

Personal ties and strong beliefs in collegiality have been ingrained over time into the culture of PubConsult. These strong personal connections and long tenure of staff made it possible that informal processes and communication structures evolved which was visible through joint lunches at the cafeteria and activities outside work.

Prior to the introduction of NWW, control was socially enacted, but it was also adapted to individual needs *via* the supervisor, who was generally aware of subordinates’ personal circumstances and could adjust expectations accordingly. However, the historically developed personalized approaches to enacting control by supervisors *via* presence are becoming more impersonal and fragmented. As described before, other, more output-oriented indicators are increasingly coming to the front. The art of control with minimal output control, achieved by exercising acts of control in symbolic ways, was previously enacted as an individually acquired expertise based on informal control practices. This personal form of control is now threatened by other methods of control. Informal control practices are at risk of being replaced by an impersonal and more norm-based and standardized mode of control, which contradicts the organizational value of respect for the individual employee and the expertise—in terms of quality—arising from the group.

Well, I recognize that there are many supervisors who are afraid they cannot maintain performance, the quality of performance – content-wise. They think that employees are sitting individually, and communication and exchange does not exist anymore, the [threat of losing the] expertise—this is a huge fear. (Supervisor)

With the shift to more output-oriented and thus more standardized modes of control, individual skills in tacit control are becoming obsolete. The controller becomes an impersonal executor of standardized control measures. Inherent is the risk that the competent supervisor with specific skills is losing control—and consequently, value—which may lead to becoming superfluous. The needed number of managers could dwindle, as technology enables only a few to do the job of many by creating the impression of constant surveillance. Thus, although there is strong support from top management to institutionalize NWW at PubConsult, this transition encountered resistance from the supervisors. Because of supervisors’ uncertainty about how to lead, they feel threatened and the need to legitimize their position in the organization. The risk of rationalization becomes discursively tangible, even in an organization like PubConsult, which is characterized as a secure work place. Supervisors become aware that their behavior, but also their thinking must change.

In the course of this working from home initiative and so forth, a new thinking about performance management and control has emerged, stringently enough. (Supervisor)

There are still many leaders who are afraid when individuals work from home. “Well, how do I control this then?” Also, there is for example a fear regarding control, a huge one, a fear of losing control. (Supervisor)

What should I say? There is a need for new behaviors or radically new thinking among the supervisors. (Supervisor)

## Discussion

The informational age (Castells, 1996; Schmiede, 2006) led to new work and organizational design concepts that have been conceptualized as NWW. NWW encompass greater freedom and flexibility for employees in terms of when and where to do work, supported by the use of information and communication technology (Demerouti et al., 2014). When employees enjoy increased freedom regarding work time and place, studies have shown the need to re-align control practices (Kurland and Cooper, 2002; Taskin and Edwards, 2007; Taskin, 2010; Sewell and Taskin, 2015), but little is known about *how* these changing control practices are negotiated in organizations. Since the data were collected prior to the COVID-19 restrictions, our study remains unbiased by the current health threat and the accompanying understanding of supervisors that employees must work remotely to protect their health.

Our analysis reveals tensions and paradoxical situations that illustrate the conflicting demands of the long-established culture of being present at the office and the newly adopted possibility of working remotely. Similar to Bjerregaard (2011), we use a praxeological approach to understand competing institutional logics and identify in our analysis three constitutive moments of transformation with regard to managerial control (how implicit becomes explicit, how collective becomes self, and how personal becomes impersonal). Based on our findings, our study makes the following contributions.

First, even though one of NWW’s fundamental objectives is increased employee autonomy, it is possible that the transition to remote working will, in fact, entail an intensification of control driven by technology (Taskin and Edwards, 2007). Research suggests that behavior control might not impair team performance, but it affects the satisfaction with the virtual team due to the lack of freedom perceived (Piccoli et al., 2004). In line with that, employees at PubConsult interpreted the installation of cameras as a symbol of control, surveillance, and mistrust, which conflicts with their organization’s long-established values of collegiality and solidarity. Unintended by management, a debate about monitoring and surveillance *via* technology was initiated in the organization. In general, supervisors are key players in driving the acceptance of digital tools and technologies (Cortellazzo et al., 2019). Obviously, the supervisors at PubConsult have not managed to persuade their subordinates about its usefulness (Zhou and Feng, 2017) as the camera discourse only circled around potential surveillance. Regardless of the initial intention, the organizational actors were aware of the technology’s affordances for implicit, hidden control, which supposedly enabled supervisors to counteract the organizational intention of “no individual performance measurement.”

Second, our analysis uncovers supervisors’ uncertainties about adapting their managerial control practices as well as their need for legitimizing their position in the organization. Despite supervisors’ awareness of the need to change, their long-established control practices were still prevailing suggesting a form of “co-option,” which is defined as the adoption of “a strategic element from another logic that retains the most important elements of its own logic” (Andersson and Liff, 2018, p. 72). Even when co-option is aimed at protecting the prevailing logic, the co-opted elements nonetheless change the prevailing logic at the individual actor level and shape institutional logics in a dynamic, iterative process. Thus, despite the management’s intention to leave their basic principles of control untouched, there is the threat of implementing output control from the immediate supervisor *via* the back door (as technology affords surveilling employees). This brings the fundamental—and often neglected—question of control to the forefront: What does doing work mean within the particular organization? This question might sound trivial at first, but our interviews revealed uncertainty about this issue and the necessity to provide clarity on it. Thus, to successfully implement NWW and adapt supervisors’ control practices, organizations must first initiate a negotiation process about what “doing work” means, since the exclusive reliance on the category of “being at work” becomes untenable in NWW. Subordinates respond to this by addressing the question about how they should signal their “doing work” showing the focus on work time for performance. However, nowadays more and more organizations transform toward asking for outputs (e.g., Voß and Pongratz, 1998), which might contradict the dominant institutional logics of PubConsult, but nonetheless seems to creep into the organization and shaping its logics. This perceived contradiction reveals that organizations need to actively address and discuss expectations on work performance—in particular when no individual output measurements are executed. A clear understanding about “doing work” allows organizations to adequately adapt (control) practices to the digital workplace and finally benefit from the advances in technology (White, 2012).

Third, motivated by a mission to protect employees’ rights, the organization was keen to organize remote work following the same long-established practices based on clearly regulated work time, with the sole difference that employees could choose to work remotely up to 20h per week. However, this practice also limited employee autonomy, as management did not trust employees to manage their own work. Moreover, it was considered a form of “necessary control” for the group of “non-workers.” In this organization, many organizational actors seem to be aware—or at least make frequent reference to—a diffuse and stigmatized group of people identified as “non-workers,” whose membership is never defined. Employees expressed concern that the introduction of remote working would allow employees from the group of “non-workers” to avoid scrutiny in the office, permitting them to be even less productive at home than at the office. Thus, offering remote working to employees challenges the organization to define how much and what kind of control should be exerted because some employees may exploit the autonomy given and reduce work engagement, which is also a common concern of supervisors (Gajendran et al., 2015; Bolino et al., 2021). Despite the commonly acknowledged group of “non-workers” at PubConsult, the majority of employees is perceived as highly committed. The organization’s management is even aware of the potential risk of self-exploitation (Voß and Pongratz, 1998) and emotional exhaustion (Schlachter et al., 2018) due to work intensification (Kelliher and Anderson, 2010). However, when being out of supervisory sight, it might be harder to distinguish between hard-working employees and “non-workers.” Due to this difficulty, remote working might even have the potential to widen the gap between both groups of employees in the absence of individualized output control. Therefore, clarity about expectations is highly needed. This might help to balance both extremes: employees who take advantage of being out of sight to work less and those who voluntarily even work harder.

Fourth, there have been calls for more research on the drivers that change institutional logics (Thornton et al., 2012), and our case shows that the implementation of NWW could be seen as potential driver. Introducing NWW might serve as an opportunity for cultural change initiatives since it potentially triggers deep changes to institutional logics and breaks up established organizational practices. However, the discourse about the camera vividly illustrates that perceptions about technology and its affordances for hidden monitoring could also backfire and represent a threat to employees. Therefore, a change toward NWW (including technology, which represents a pre-requisite for NWW) needs to be implemented with great care and combined with active information measures as the usage and purpose of new technology must be legitimized in the organization. Finally, it is worth noting that organizational actors at PubConsult were accustomed to raising concerns and questioning the implementation of change. Because the underlying organizational mission of PubConsult was to protect employees’ rights, PubConsult’s own employees did not fear any negative consequences by speaking up. This might also explain why issues such as the fear of losing the job due to inappropriate social media behavior (i.e., doocing; Cortini and Fantinelli, 2018) were not addressed at all in the interviews. Due to the high job security at PubConsult, the case presents a unique opportunity to gain insight into potentially hidden and suppressed dynamics in similar organizational structures where such candor might not exist. Thus, we argue that the findings specific to PubConsult can also speak to a wider debate on NWW in other organizations that refrain from individual output control.

Interestingly, also concerns about an invasion of the private sphere—with respect to PubConsult’s camera debate—were never addressed in the interviews. Social ties among colleagues were very high at PubConsult, and employees were accustomed to knowing about their colleagues’ private lives. This may have contributed to employees’ conspicuous lack of resistance to granting visual access to their homes. This assumption must be tested by investigating other, similar organizations in future studies. Examining the long-term aspects of the adaptation process to “doing work” and its effect on managerial control practices may also be an interesting route for further research.

## Practical Implications For the Post-Covid-19 Period

Although the trend toward NWW has begun prior to COVID-19, it was further accelerated by the outbreak of the pandemic. Without much preparation, many workers rapidly had to adapt their working practices and move their work space into their homes. Due to the pandemic, remote work has become “the new normal in working life” (Bjursell et al., 2021) and it is not a work design element offered only by early adopters anymore (*cf*., Daniels et al., 2001). Despite all encountered challenges, employees also cherished the new flexibility of remote work and wish to continue—at least occasionally—to work remotely after COVID-19 (Beno and Hvorecky, 2021; Fana et al., 2021).

The pandemic has very likely fundamentally challenged the institutional logics with regard to managerial control. Wang et al. (2021) have studied the challenges experienced by Chinese remote workers during the pandemic and showed that monitoring when working remotely had a negative effect on employees because it resulted in more home–work conflicts. Pre-COVID-19 research shows that monitoring *sensu* behavior control relates to trust decline in virtual teams as members’ failure to uphold obligations will be more easily detected (Piccoli and Ives, 2003). However, trust is considered as a key aspect for effective remote work (Lengen et al., 2021). On the other hand, the study of Wang et al. (2021, p. 29) also suggests that monitoring can “help them [employees] to cope with procrastination and to concentrate on their core tasks.” The relationship between control and trust has been controversially discussed in organization research (Weibel, 2007): On the one hand, control and trust constitute functional equivalents and are perceived as interchangeable. On the other hand, control and trust might also amplify each other; e.g., employees working hard might prefer supervisors who monitor work performance and sanction misbehavior, which then establishes fairness and fosters trust in the supervisor. Our results about the “non-workers” point toward the latter perspective. Informants acknowledged the existence of social loafers and actively distinguished themselves from this group fearing that their supervisor might not be able to see their work engagement when working at home. Thus, communicating effectively with others when working remotely is essential (Piccoli et al., 2004; Galanti et al., 2021). This becomes even more important considering that well-implemented controls can even create trust in the organization (Weibel et al., 2016). However, what forms of control and whether it is perceived as legitimate depend on the prevailing institutional logics. Therefore, organizations have to reflect on their prevailing institutional logics and to re-evaluate their remote working practices in order to prepare for the post-COVID-19 period. We expect that pre-pandemic discourses on managerial control practices will surface again the longer we continue in the “new normal.”

## Conclusion

Understanding the potential dynamics underlying the introduction of remote working may help to guide supervisors in adapting their managerial control practices to the new realities. Our findings demonstrate that the success of introducing NWW depends on taking into account prevailing rules, norms, and cultural cognitions. Supervisors in organizations with cultures that place high value on physical presence and which lack individual output measures are likely pressured to adapt managerial control. Inscribed managerial control practices in public bureaucracies that refrain from individual output control are in conflict with NWW. The case is illustrative of ongoing discourses about managerial control triggered by NWW in an organization embedded in a stable and secure environment and demonstrates the relevance of considering the underlying institutional logics before implementing NWW. The case also demonstrates the necessity of supporting supervisors as they re-define their control practices. Identifying the prevailing institutional logics helps to create a shared understanding of leadership in NWW and might even help to uncover dysfunctional work processes.

## Data Availability Statement

The datasets presented in this article are not readily available because interview transcripts contain identifiable information which conflicts with the assured anonymity of the interviewees. Requests to access the data should be directed to martina.hartner-tiefenthaler@tuwien.ac.at.

## Ethics Statement

Ethical review and approval was not required for the study on human participants in accordance with the local legislation and institutional requirements. Written informed consent for participation was not required for this study in accordance with the national legislation and the institutional requirements.

## Author Contributions

MH-T: planning and execution of study, conduction of all interviews, in-depth analysis and conclusions, writing - original draft preparation and review, and editing. MG: in-depth analysis and conclusions, writing - original draft preparation and review, and editing. CG: conceptual ideas, interpretation of data, critical revisions, and writing – original draft preparation. SK: contributed to the conceptual ideas, the interpretation of data, and critical revisions. All authors contributed to the article and approved the submitted version.

## Conflict of Interest

The authors declare that the research was conducted in the absence of any commercial or financial relationships that could be construed as a potential conflict of interest.

## Publisher’s Note

All claims expressed in this article are solely those of the authors and do not necessarily represent those of their affiliated organizations, or those of the publisher, the editors and the reviewers. Any product that may be evaluated in this article, or claim that may be made by its manufacturer, is not guaranteed or endorsed by the publisher.

## Footnotes

^1^To protect the organization’s anonymity, we cannot provide further detailed information of the organization.
